# Development of an Efficient Somatic Embryogenesis Protocol for *Carica papaya* L. Var. TNAU Papaya CO 8 on Different Basal Media

**DOI:** 10.3390/plants15060893

**Published:** 2026-03-13

**Authors:** Shalini Chandrasekar, Kavitha Chinnasamy, Ganga Mathian, Krish K Kumar, Babu Rajendra Prasad, Manoranjitham S. Karuppannan, Selvaraju Kanagarajan, Saraladevi Muthusamy

**Affiliations:** 1 Department of Fruit Science, Horticultural College and Research Institute, Tamil Nadu Agricultural University, Coimbatore 641003, Tamil Nadu, India; shalini.sbjv@gmail.com; 2 Turmeric Research Centre, Tamil Nadu Agricultural University, Bhavanisagar 638451, Tamil Nadu, India; 3 Department of Floriculture & Landscape Architecture, Horticultural College and Research Institute, Tamil Nadu Agricultural University, Coimbatore 641003, Tamil Nadu, India; ganga.m@tnau.ac.in; 4 Department of Plant Biotechnology & Molecular Biology, Centre for Plant Molecular Biology & Biotechnology, Tamil Nadu Agricultural University, Coimbatore 641003, Tamil Nadu, India; kumarbiotech@gmail.com; 5 Department of Crop Physiology, Tamil Nadu Agricultural University, Coimbatore 641003, Tamil Nadu, India; prasadvenugopal@gmail.com; 6 Department of Plant Pathology, Tamil Nadu Agricultural University, Coimbatore 641003, Tamil Nadu, India; manoranjitham.k@gmail.com; 7 Department of Plant Breeding, Swedish University of Agricultural Sciences, 23422 Lomma, Sweden; selvaraju.kanagarajan@slu.se

**Keywords:** *Carica papaya*, immature zygotic embryos, embryogenic callus, somatic embryogenesis, regeneration, micropropagation, plant growth regulators

## Abstract

Papaya (*Carica papaya* L.) is a highly cross-pollinated crop that exhibits considerable genetic variability when propagated through seeds, resulting in non-true-to-type progeny. Therefore, the development of an efficient in vitro regeneration system is essential for large-scale clonal propagation of elite cultivars. In the present study, a highly efficient and reproducible somatic embryogenesis protocol was developed for *C. papaya* var. TNAU Papaya CO 8 using immature zygotic embryos as explants. This study provides the first comprehensive comparative evaluation of three basal media, viz., Murashige and Skoog Medium, N6 Medium, and Woody Plant Medium, for somatic embryogenesis and plant regeneration in this variety, along with the optimization of polyamine-enriched media for enhanced plantlet recovery. The embryogenic potential of explants was assessed across different stages, including callus induction, somatic embryo development, plant regeneration, shoot elongation, rooting, and acclimatization. Maximum callus induction (81.96%) was observed on half-strength MS medium supplemented with 2,4-Dichlorophenoxyacetic acid under dark conditions, followed by ½ N6 (63.00%) and ½ WPM (58.02%). Somatic embryo initiation was highest on ½ MS medium containing 2.0 mgL^−1^ 2,4-D (77.82%). Somatic embryos developed through distinct globular, heart, torpedo, and cotyledonary stages. Embryo maturation was significantly enhanced on MS medium supplemented with abscisic acid, polyethylene glycol, benzylaminopurine, and proline. The highest plantlet regeneration (85.02%) was achieved on MS medium enriched with putrescine, whereas comparatively lower regeneration was recorded on N6 (75.99%) and WPM (57.97%). Shoot elongation was significantly improved by supplementation with gibberellic acid (1.0 mgL^−1^). Root induction was optimal on half-strength MS medium containing Indole-3-butyric acid, 1-Naphthaleneacetic acid, phloroglucinol, and activated charcoal, resulting in well-developed roots. Regenerated plantlets were successfully acclimatized in a cocopeat–vermicompost substrate with a survival rate of 74.01%. The optimized protocol provides a reliable and efficient system for large-scale clonal propagation and offers promising applications in genetic transformation and commercial production of papaya var. TNAU papaya CO 8.

## 1. Introduction

Papaya (*Carica papaya* L.), a member of the family Caricaceae, is a commercially important fruit crop, cultivated extensively across tropical and subtropical regions worldwide. The crop has gained commercial importance due to its high productivity, year-round fruiting ability, rich nutritional composition, and multifaceted medicinal properties [1]. Papaya, which originated in Southern Mexico and Central America, was later domesticated across Asia, Africa, Oceania, and North America; at present, the major papaya-producing countries include India, Brazil, Mexico, Indonesia, and Nigeria [2,3,4]. Owing to its rich nutritional composition, papaya is an excellent source of vitamins (A, B, C, E, and K), carotenoids (zeaxanthin, lycopene, and lutein), and essential minerals such as calcium, magnesium, copper, and potassium [4]. Additionally, it possesses diverse pharmacological properties, including antioxidant, anticancer, wound-healing, and digestive health benefits [5,6]. Beyond its use as a dessert fruit, papaya has substantial industrial significance owing to the extraction of papain, a proteolytic enzyme used in meat tenderization, preparation of chewing gum, pre-shrinking of wool, degumming of natural silk, and in pharmaceutical, cosmetic, textile, brewing, and leather industries [7].

Papaya is conventionally propagated through seeds; however, high genetic heterozygosity and a polygamous nature result in considerable variability in yield, fruit quality, and responses to pest and disease infestations [8,9]. Vegetative propagation techniques such as cuttings and grafting are comparatively less effective and economically less viable in papaya due to poor rooting ability, low multiplication rates, and reduced survival of propagules. In addition, these methods can further spread sap-transmissible Papaya Ring Spot Virus (PRSV) disease [10]. Therefore, in vitro propagation techniques offer an effective alternative to overcome the limitations of the aforementioned propagation methods in papaya and facilitate large-scale, rapid, and disease-free plant production [11,12,13,14]. Several in vitro regeneration approaches, including axillary shoot proliferation [15,16] and somatic embryogenesis (SE), have been successfully employed in papaya [17,18,19]. In direct embryogenesis, embryos are inducted directly from the explant, whereas induction of new somatic embryos from primary embryos or embryogenic calli, with or without an intervening callus phase, was achieved in the latter [20,21,22,23]. Somatic embryogenesis, in addition to plantlet production, serves as an effective system for genetic transformation, mutation studies, and synthetic seed production, and plays a crucial role in papaya crop improvement [24,25].

Papaya is considered a recalcitrant species for in vitro culture, with somatic embryo induction being highly genotype-dependent and often associated with problems such as callus browning, low embryogenic frequency, and abnormal embryo formation [26]. The success of somatic embryogenesis largely depends on factors such as the type and physiological stage of the explants, composition of the basal medium, and the concentration of plant-growth regulators (PGRs) [27,28,29]. Among these factors, the choice of basal medium plays a critical role in determining morphogenic responses. Basal media such as Murashige and Skoog Medium [30], Woody Plant Medium [31], and N6 Medium [32] differ considerably in their macro- and micronutrient compositions, nitrogen sources, and salt concentrations, which directly influence tissue growth, callus quality, and embryogenic potential. While MS medium is widely used for a broad range of dicotyledonous species, WPM and N6 media are reported to support embryogenic responses in certain woody and recalcitrant plant species by maintaining balanced nutrient availability and reducing physiological stress. However, a systematic comparative evaluation of these nutritionally diverse basal media for somatic embryogenesis in papaya remains limited. Plant-growth regulators also play a crucial role in regulating cell division, differentiation, and morphogenesis during in vitro culture. Auxins are generally associated with callus induction and root formation, whereas cytokinins promote shoot regeneration and embryogenic differentiation [33,34]. Nevertheless, the conversion of somatic embryos into viable plantlets remains a major constraint in papaya tissue culture due to low germination rates, abnormal root development, and somaclonal variation that can affect plant stability and field performance [35,36]. These challenges highlight the need for developing a reliable and reproducible regeneration protocol.

TNAU Papaya CO 8 is a high-yielding, red-pulped, dioecious variety commercially cultivated in India for its higher productivity and relatively better field tolerance to Papaya Ringspot Virus (PRSV) compared with gynodioecious varieties [37]. Since the variety is propagated through seeds, early identification of plant sex is not possible until flowering. Consequently, farmers usually plant four to six seedlings per pit and remove excess male plants during flowering, resulting in increased production costs and management difficulties. Furthermore, maintaining genetic purity is challenging due to the crop’s high heterozygosity, and careful selection of male and female plants for sib-mating is essential. Therefore, the present study was undertaken to develop an efficient and reproducible somatic embryogenesis protocol for papaya var. TNAU Papaya CO 8 using immature zygotic embryos as explants. A comparative evaluation of three basal media—Murashige and Skoog Medium, Woody Plant Medium, and N6 Medium—was carried out to determine their effectiveness in inducing embryogenic callus and promoting somatic embryo development. The study further aimed to evaluate the influence of different plant-growth regulators on embryogenic response, plant regeneration, and rooting, and to establish an optimized protocol for efficient clonal propagation of this commercially important papaya variety.

## 2. Results

### 2.1. Callus Induction and Embryogenesis Percentage (%)

The immature zygotic embryos cultured on half-strength MS, WPM, and N6 media supplemented with different concentrations of 2,4-D and picloram initiated callus formation after 18–29 days of incubation under dark conditions (Table 1). No morphogenic response was observed in control treatments lacking growth regulators, irrespective of the basal medium. The cultures were maintained for 6 weeks, during which subculturing was performed at 15-day intervals to promote callus proliferation. In all three basal media, embryos cultured on 2,4-D-containing media exhibited earlier and more pronounced callus induction than those treated with picloram. Among the basal media, ½ MS medium showed the early callus induction, requiring 18.00 days at 2.5 mgL^−1^ 2,4-D, which is significantly earlier than ½ N6 (21.26 days) at 2.5 mgL^−1^ 2,4-D. In comparison, ½ WPM recorded the longest duration (23.19 days) at the same concentration with creamy white friable callus (Table 1). Picloram treatments consistently delayed callus initiation, with maximum induction time observed on ½ WPM supplemented with 1.0 mgL^−1^ picloram (28.93 days).

**Figure 1:**
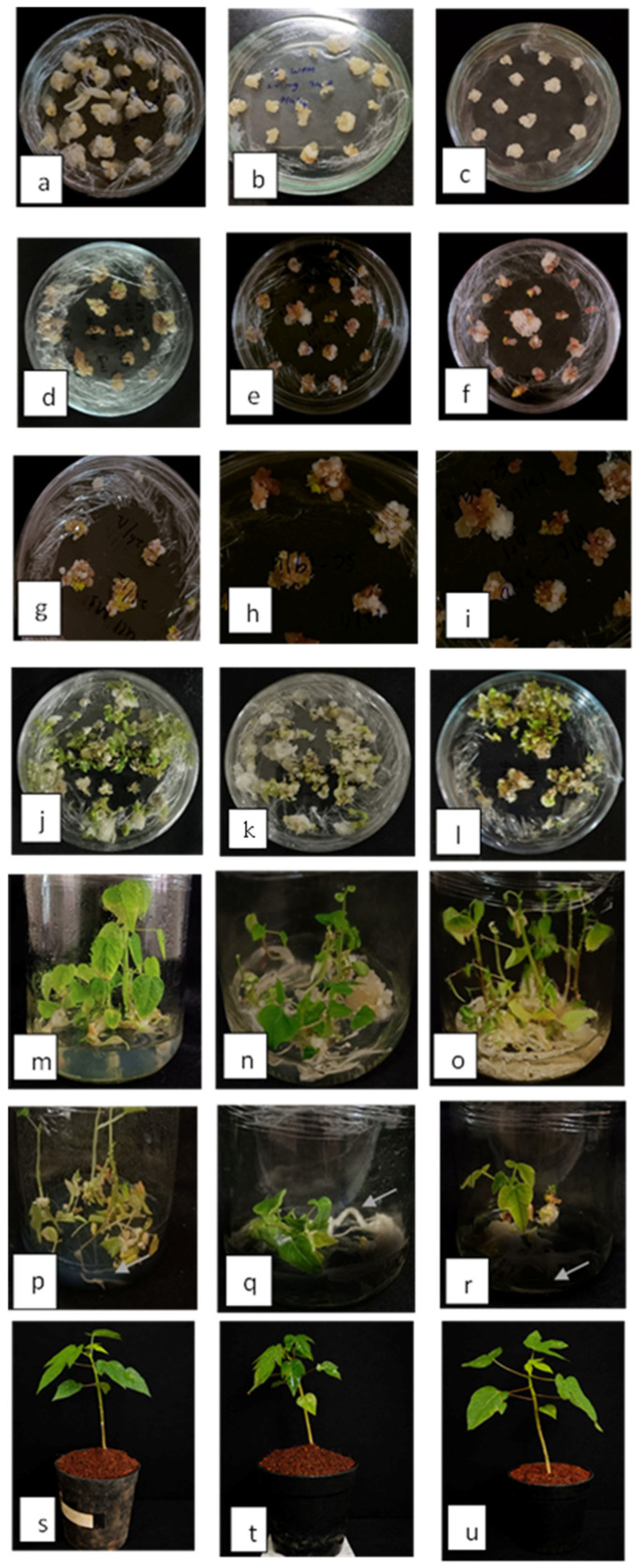
(**a**–**c**) Callus formation on MS, WPM, and N6 media containing 2.5 mgL^−1^ 2,4-D respectively; (**d**–**f**) callus maturation on MS, WPM, and N6 media, respectively; (**g**–**i**) clusters of germinating cotyledonary embryo on MS, WPM, and N6 media, respectively; (**j**–**l**) plumule development from cotyledonary embryos of MS, WPM, and N6 media, respectively; (**m**–**o**) trilobed leaves with elongated shoots on 1.0 mgL^−1^ GA_3_ on MS, WPM, and N6 media; (**p**–**r**) plantlets with root initials on MS, WPM, and N6 media, respectively; (**s**–**u**) well-developed shoot and root systems from different basal media, maintained in pots containing cocopeat and vermicompost before field transplanting.

Callus induction percentage was highest on ½ MS medium, reaching 81.96% and 80.20% at 2.0 and 2.5 mgL^−1^ 2,4-D, respectively. In comparison, the maximum callus induction on ½ N6 was 63.00% (2.5 mgL^−1^ 2,4-D), while ½ WPM recorded the minimum of 58.02%. Picloram induced moderate callus formation across all media. Embryogenesis percentage followed a similar trend (Figure 1a–c). The maximum embryogenesis (77.82%) was obtained on ½ MS medium supplemented with 2.0 mgL^−1^ 2,4-D, followed by 72.11% at 2.5 mgL^−1^. In ½ N6, maximum embryogenesis percentage (72.15%) was recorded at 2.0 mgL^−1^, whereas ½ WPM showed comparatively lower embryogenic conversion, with a maximum of 68.01% at 2.5 mgL^−1^.

### 2.2. Maturation of Somatic Embryos

**Figure 2:**
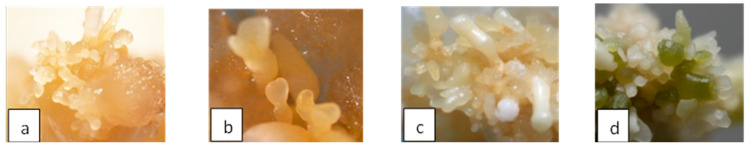
Different stages of somatic embryo development during somatic embryogenesis: (**a**) Globular; (**b**) heart; (**c**) torpedo; and (**d**) cotyledonary.

After callus initiation, the developing calli were periodically examined under a stereomicroscope (Zeiss Stemi DV4, Jena, Germany) to assess their morphological and developmental stages (Figure 2). Once embryogenic stages, such as the globular (Figure 2a) and heart (Figure 2b) stages, were confirmed, the calli were transferred to maturation medium.

**Figure 3:**
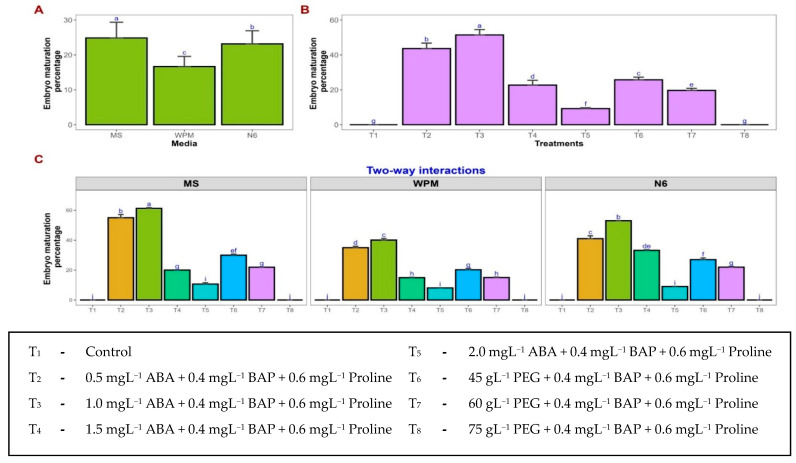
Effect of ABA, PEG, BAP, and proline on embryo maturation percentage (%) on MS, WPM, and N6 media. Impact of basal media and treatments on somatic embryo maturation percentage. Mean comparisons were performed for media (**A**), treatments (**B**), and media × treatments interaction (**C**). Values represent the mean of three replicates, and error bars indicate the standard error. Means followed by the same letter are not significantly different according to Tukey’s test (*p* < 0.05).

Embryo maturation was significantly affected by the culture medium, treatments, and their interaction. No embryo maturation was observed in the control across all three media. Among the media tested, MS medium had the highest percentage of embryo maturation, followed by N6 and WPM. In MS medium, the highest embryo maturation (61.25%) was observed with 1.0 mgL^−1^ ABA + 0.4 mgL^−1^ BAP + 0.6 mgL^−1^ proline, whereas lower (0.5 mgL^−1^; 55.02%) and higher ABA concentrations resulted in reduced maturation (Figure 1d–f). A similar response was observed in WPM and N6 media, where the same ABA concentration (1.0 mgL^−1^) produced 40.07% and 53.00% embryo maturation, respectively (Figure 3). Increasing ABA concentration beyond 1.0 mgL^−1^ significantly decreased embryo maturation in all media, with the lowest values recorded at 2.0 mgL^−1^ ABA (8.13 to 10.65%). PEG-induced osmotic treatments showed moderate embryo maturation, with 45.0 gL^−1^ PEG producing 20.24–29.98% across media, whereas higher PEG levels (75.0 gL^−1^) completely inhibited embryo maturation.

### 2.3. Somatic Embryo Regeneration and Shoot Elongation

The influence of various concentrations of putrescine and phloroglucinol on somatic embryo germination is presented in Table 2. Mature cotyledonary embryos were cultured under different treatment levels to assess their regeneration response. Among the treatments, 2.0 mgL^−1^ putrescine in MS medium recorded the highest embryo regeneration percentage (85.02%), with the early leaf emergence (16.00 days) and the maximum shoot length (1.31 cm). Similar patterns were observed in N6 medium, where 2.0 mgL^−1^ putrescine also resulted in improved regeneration (75.99%) and shoot elongation (1.00 cm). On WPM, regeneration responses were comparatively lower, with the highest regeneration (57.97%) observed at 2.0 mgL^−1^ putrescine, along with moderate shoot length (0.70 cm) and delayed leaf emergence (19.00 days) (Figure 1g–i). In contrast, higher concentrations of putrescine (≥2.5 mgL^−1^) led to a gradual decline in regeneration efficiency and shoot growth, while lower concentrations (1.0 mgL^−1^) delayed leaf emergence. Treatments with phloroglucinol (5.0 mgL^−1^ and 10.0 mgL^−1^) did not induce any regeneration or shoot formation across all media.

**Figure 4:**
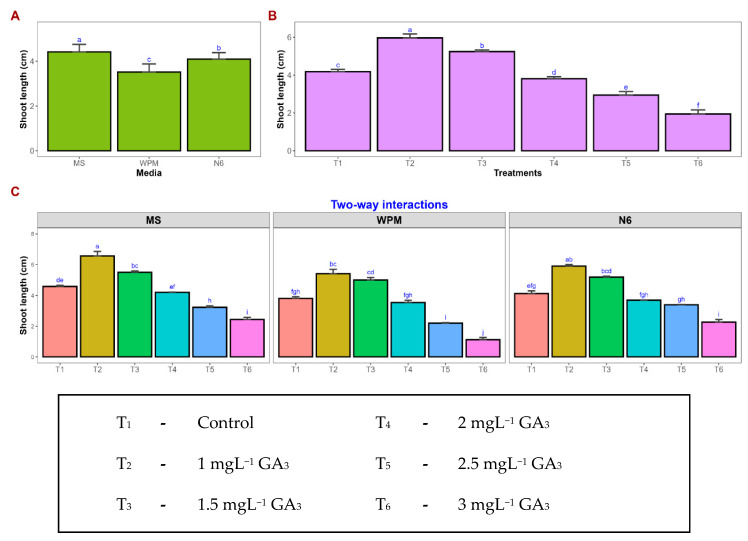
Response of shoots to gibberellic acid treatment on shoot elongation. Impact of basal media and treatments on shoot elongation. Mean comparisons were performed for media (**A**), treatments (**B**), and media × treatments interaction (**C**). Values represent the mean of three replicates, and error bars indicate the standard error. Means followed by the same letter are not significantly different according to Tukey’s test (*p* < 0.05).

To promote shoot elongation, the regenerated shoots were transferred to media enriched with different concentrations of GA_3_. Among the treatments, MS medium supplemented with 1.0 mgL^−1^ GA_3_ exhibited the maximum shoot length (6.57 cm), followed by N6 (5.92 cm) and WPM (5.41 cm) at the same concentration (Figure 1m–o). An increase in GA_3_ concentration beyond 1.5 mgL^−1^ resulted in a gradual decrease in shoot length across all media, indicating that higher levels of GA_3_ may inhibit elongation. The shortest shoot length (1.12 cm) was observed in WPM with 3.0 mgL^−1^ GA_3_ (Figure 4).

### 2.4. Root Induction and Acclimatization

The rooting performance of shoots obtained from GA_3_-induced elongated shoots of papaya after four weeks of culture was significantly influenced by basal media and treatments, as presented in Table 3. The effects of various concentrations of IAA, IBA, and NAA, combined with phloroglucinol and activated charcoal, were evaluated. Significantly higher rooting response was obtained on ½ MS medium, followed by ½ N6 medium; ½ WPM showed the lowest rooting response. In ½ MS medium, the highest rooting percentage (75.01%), number of roots (6.99), minimum days taken for rooting (19.02 days), and the longest root length (3.80 cm) were recorded in the treatment containing 1.0 mgL^−1^ IBA + 0.5 mgL^−1^ NAA + 1.0 mgL^−1^ PG + 1.0 gL^−1^ AC. In ½ N6 medium, the same treatment resulted in 72.01% rooting, with an average of 6.09 roots per shoot and a mean root length of 3.40 cm, while root initiation occurred within 19.99 days. In ½ WPM, the highest rooting percentage (69.00%), 21.02 days to rooting, and 5.50 roots with a root length of 3.19 cm were also observed with the IBA + NAA + PG + AC treatment, though the overall response was lower than in ½ MS and ½ N6. Across all media, treatments supplemented with activated charcoal performed better than those without it, whereas control treatments failed to induce rooting. The interaction effect between media and treatments indicated that ½ MS medium supplemented with IBA, NAA, phloroglucinol, and activated charcoal was the most effective combination for rooting (Figure 1p–r).

**Figure 5:**
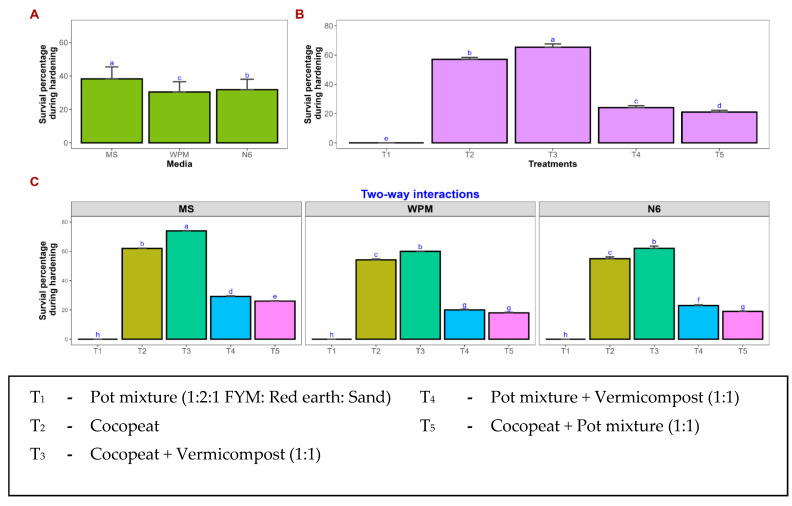
Survival percentage of hardened plantlets derived from different basal media. Impact of basal media and treatments on survival percentage. Mean comparisons were performed for media (**A**), treatments (**B**), and media × treatments interaction (**C**). Values represent the mean of three replicates, and error bars indicate the standard error. Means followed by the same letter are not significantly different according to Tukey’s test (*p* < 0.05).

After two months of hardening, the survival percentage was recorded (Figure 5). Among the hardening media, cocopeat and vermicompost (1:1) (T_3_) resulted in significantly higher survival percentage across all basal media, with maximum survival observed in MS-derived plantlets (74.01%), followed by N6 (62.08%) and WPM (59.98%) (Figure 1s–u). Hardening media with cocopeat alone (T_2_) also supported better acclimatization, resulting in moderate survival rates of 62.02%, 55.00%, and 54.21% in MS, N6, and WPM media, respectively. The conventional pot mixture containing FYM, red earth, and sand (T_1_) failed to support plantlet establishment, resulting in complete mortality across all culture media.

## 3. Discussion

In the present study, a comparative assessment of media composition with growth regulators was conducted during callus induction, somatic embryo development and maturation, somatic embryo regeneration, in vitro rooting, and acclimatization in the papaya variety TNAU Papaya CO 8. Somatic embryogenesis in papaya is a strongly genotype-dependent process, with considerable variability among cultivars under in vitro conditions. In the present investigation, the cultivar TNAU Papaya CO 8 exhibited a highly efficient and reproducible embryogenic response when cultured under optimized conditions. The consistent formation of embryogenic calli, successful somatic embryo development, and subsequent plant regeneration observed in this study indicate that this genotype possesses a strong inherent capacity for in vitro morphogenesis. Such genotype-specific responses have been previously documented in papaya, where differences in endogenous hormonal balance, cellular totipotency, and metabolic activity significantly influence embryogenic potential. The reliable response obtained in the present study suggests that the developed protocol is well-suited for this cultivar and may be effectively utilized for large-scale clonal propagation. Among the media, MS medium consistently supported superior callus induction frequency, embryogenic callus quality, and regeneration efficiency compared with WPM and N6 medium [38]. The enhanced performance of MS medium may be attributed to its higher total salt concentration and balanced macro- and micronutrient composition, particularly its elevated nitrogen content, which collectively enhances cellular differentiation and morphogenesis during embryogenesis [39]. In addition, the presence of specific compounds, such as potassium nitrate, cobalt chloride, and potassium iodide, enhances osmotic balance and nutrient uptake, thereby facilitating better callus induction, somatic embryo formation, and overall regeneration efficiency in papaya [40,41]. However, the sucrose content in WPM is lower compared to MS and N6 media, and this difference may also have influenced the overall growth response observed in the present study.

Callus induction represents a critical initial step in somatic embryogenesis, as it determines the embryogenic competence of cultured tissues. The highest percentage of callus formation (81.96%) was observed in ½ MS medium supplemented with 2.0 mgL^−1^ 2,4-D, compared with ½ WPM and ½ N6 media and other treatment combinations. This result indicates that MS medium provided a more favorable nutrient balance for callus induction, while 2,4-D at optimal concentration effectively promoted cellular dedifferentiation and the initiation of embryogenic calli. The role of 2,4-D in inducing somatic embryogenesis from immature zygotic embryos has been well established in papaya [42,43,44,45,46]. Although earlier reports suggested that higher concentrations of 2,4-D could enhance somatic embryo production, the present findings demonstrate that a lower concentration (2.0 mgL^−1^) is sufficient to achieve high embryogenic callus formation in this genotype. In contrast, higher concentrations led to callus browning and a reduced embryogenic response, suggesting that excessive levels of 2,4-D may exert a toxic or inhibitory effect on tissue development. Furthermore, prolonged exposure or excessive levels of 2,4-D could increase the risk of somaclonal variation in regenerated plantlets, emphasizing the importance of optimizing auxin levels for stable in vitro propagation [47]. Treatments containing picloram exhibited delayed callus initiation, indicating that 2,4-D was more effective for inducing embryogenic competence in this cultivar [48,49]. Hence, optimizing auxin concentration is crucial for achieving effective callus induction while maintaining genetic stability during in vitro propagation.

Maturation is a vital phase in somatic embryogenesis, encompassing key physiological events such as cell enlargement, tissue differentiation, and the accumulation of reserve substances necessary for subsequent germination and plant regeneration. In the present study, supplementation of the maturation medium with abscisic acid (ABA) and proline significantly improved somatic embryo development and maturation. However, the present observations differ from previous reports that demonstrated successful maturation of papaya somatic embryos in the absence of exogenously supplied growth regulators [50]. Consistent with previous reports [51], the present study demonstrated that supplementation of the culture medium with abscisic acid (ABA) significantly enhanced somatic embryo production and maturation. ABA is widely recognized as a key regulator of embryogenesis and embryo maturation [52,53,54]. It plays a crucial role in promoting the synthesis of storage proteins and lipids, preventing precocious germination, and inducing the accumulation of late embryogenesis abundant (LEA) proteins that are associated with desiccation tolerance [55,56]. Furthermore, exogenous application of ABA may restrict excessive tissue proliferation and promote embryo differentiation through the modulation of nucleotide biosynthesis pathways [57]. In the present study, the addition of 1.0 mgL^−1^ ABA to the maturation medium resulted in optimal embryo maturation. However, higher concentrations of ABA led to desiccation and browning of the embryogenic calli, indicating possible inhibitory effects at elevated levels. The combined application of ABA and 6-benzylaminopurine (BAP) proved effective in promoting somatic embryo maturation, suggesting a synergistic interaction between these growth regulators during the later stages of embryogenic development. In addition, supplementation with proline further enhanced embryo maturation, likely by supporting embryogenic competence and cellular differentiation. Previous studies have also highlighted the beneficial role of proline in stimulating embryogenic callus proliferation, particularly in grasses and cereal crops [58,59,60]. These findings are in agreement with earlier reports [17], which indicated that optimal ABA concentrations promote embryo maturation, whereas excessive levels negatively affect tissue viability.

The regeneration of somatic embryos into complete plantlets represents a key step in establishing an efficient plant regeneration system. In the present study, the role of putrescine in promoting somatic embryo regeneration and germination was evaluated by incorporating different concentrations (1.0–3.0 mgL^−1^) of putrescine, along with phloroglucinol (5.0 and 10.0 mgL^−1^), into the embryo regeneration media of MS, WPM, and N6. During somatic embryo maturation, the globular, heart, torpedo, and cotyledonary developmental stages were clearly observed under a stereomicroscope, and the cotyledonary-stage embryos were transferred to regeneration media. Supplementation with putrescine stimulated cell proliferation and facilitated the transition from the cotyledonary stage to germination, indicating improved embryogenic competence and physiological quality of the embryos. Putrescine, an aliphatic polyamine, plays a vital role in plant growth and development, and is actively involved in a wide range of biological processes, including cell division, morphogenesis, secondary metabolism, chromatin organization, protein synthesis, and protein–DNA interactions [61,62,63]. In the present study, supplementation with 2.0 mgL^−1^ putrescine resulted in the highest regeneration percentage (85.02%) and successful development of somatic embryos into complete plantlets. These findings are consistent with earlier reports in wheat [64] and sweet pepper [65]. In contrast, phloroglucinol did not promote somatic embryo germination or subsequent plantlet regeneration, suggesting that putrescine exerts a more pronounced promotive effect on somatic embryo germination and plantlet regeneration under in vitro conditions. Notably, substantial regeneration was observed in the control treatments across all three basal media, with regeneration frequencies of 75.18% in MS, 50.23% in WPM, and 60.00% in N6. This response may be attributed to the inherent nutrient composition of the basal media, indicating that hormone-free media can adequately support somatic embryo germination and plantlet regeneration, as also reported previously in papaya [17].

Following embryo germination, efficient shoot elongation is necessary for the development of well-formed plantlets suitable for rooting and acclimatization. In the present study, for shoot elongation, germinated embryos were transferred to elongation media based on MS, WPM, or N6 formulations, supplemented with varying concentrations of GA_3_ (1.0–3.0 mgL^−1^). The addition of Gibberellic acid to the elongation medium significantly improved shoot growth, with the best elongation observed at 1.0 mg L^−1^ GA_3_ in MS medium. These results are consistent with earlier reports in the papaya hybrid ‘Surya,’ where supplementation of the proliferation medium with 1.0 mgL^−1^ GA_3_ promoted superior micro-shoot elongation [66]. Similar effects have also been documented in other studies [67,68], further confirming the positive role of optimal GA_3_ concentrations in enhancing shoot elongation. Gibberellic acid is widely known to promote cell elongation and stem growth, thereby facilitating the development of elongated shoots capable of further rooting.

Root induction is a critical, often limiting step in papaya micropropagation. Successful root initiation at the basal end of in vitro-regenerated shoots is essential for the subsequent acclimatization of plantlets. Earlier studies have demonstrated the effectiveness of auxins, such as NAA, IBA, and IAA, in promoting root formation [69]. In the present investigation, a lower concentration of IBA (1.0 mgL^−1^) was found to be most effective for root induction. The maximum rooting response was achieved with the combined application of 1.0 mgL^−1^ IBA, 0.5 mgL^−1^ NAA, 1.0 mgL^−1^ phloroglucinol, and 1.0 gL^−1^ activated charcoal. Activated charcoal and phloroglucinol markedly enhanced rooting efficiency, likely by improving medium aeration, providing a partially darkened environment, and adsorbing inhibitory phenolic compounds and metabolic residues. These findings are in agreement with earlier reports [70,71]. Additionally, phloroglucinol is known to function as an auxin synergist, thereby enhancing auxin-mediated root induction [72].

The final stage of the regeneration process involves acclimatization, during which in vitro-raised plantlets adapt to the external environment, and the choice of potting mixture plays a decisive role. During this transition, plantlets undergo a metabolic shift from heterotrophic to autotrophic nutrition, rendering them highly sensitive to external environmental factors. In the present study, plantlets were successfully acclimatized using a potting mixture comprising cocopeat and vermicompost. The favorable physicochemical properties of cocopeat, along with the nutrient-rich nature of vermicompost, likely contributed to improved plant establishment and growth. These findings are similar to earlier reports on *Bambusa balcooa* [73], banana var. Elakki [74], and *Garcinia indica* [75]. However, the complete mortality observed in the potting mixture (1:2:1 FYM: red earth: sand) may be attributed to its unfavorable substrate characteristics, which could have affected aeration and moisture balance during the acclimatization stage.

Overall, the results of this study demonstrate the successful development of a complete and efficient somatic embryogenesis-based regeneration system for *C. papaya* var. TNAU Papaya CO 8. The protocol integrates all critical stages of in vitro development, including embryogenic callus induction, embryo maturation, somatic embryo regeneration, shoot elongation, root induction, and acclimatization. The high regeneration efficiency and survival rates observed highlight the potential of this system for rapid clonal propagation and large-scale production of elite papaya planting material.

## 4. Materials and Methods

### 4.1. Planting Material and Disinfection

**Figure 6:**
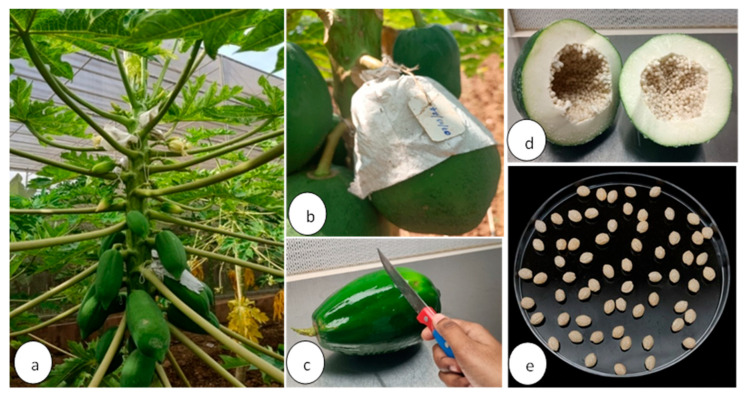
(**a**) Sib-mated fruits in the field; (**b**) sib-mated and bagged fruits; (**c**) cutting of fruits under aseptic conditions; (**d**) fruits were cut open aseptically, and the seeds were collected; (**e**) aseptic removal of sarcotesta.

The mother block of papaya (*C. papaya* L.) var. TNAU Papaya CO 8 was maintained in the College Orchard, Horticultural College and Research Institute, Tamil Nadu Agricultural University (TNAU), Coimbatore. True to type, healthy and disease-free male and female plants were identified and tagged after flowering. Sib-mating was done in the tagged mother plants, and immature fruits (90 to 100 days after fruit set) were harvested (Figure 6a,b) and carried to the tissue culture laboratory, Department of Plant Biotechnology and Molecular Biology, CPMB&B, TNAU, Coimbatore, for further study. The fruits were cleaned under running tap water to remove dirt, then surface-sterilized by immersing them in a 1.25% (*v*/*v*) sodium hypochlorite solution supplemented with two drops of Tween-20 for 60 min, followed by three rinses with sterile distilled water. Under aseptic conditions, the fruits were cut open cross-sectionally, and the seeds were collected in sterile 100 mm × 15 mm Petri dishes (Himedia, Mumbai, India) (Figure 6c–e). The seeds were cream-colored with a firm texture, and the embryos were visually identified as being in the torpedo-to-early cotyledonary stage, based on characteristic morphology of the embryonic axis and developing cotyledons under a stereomicroscope (Zeiss Stemi DV4, Jena, Germany) in a laminar air flow chamber (MARK, Mumbai, India). Seed dissection and excision of immature zygotic embryos from seeds were also performed, and to prevent dehydration during prolonged dissection, a portion of the seeds was temporarily stored at 4 °C until further use. Before embryo excision, immature seeds were washed thoroughly with sterile distilled water, followed by sterile distilled water containing Tween-20 for 1 min, then rinsed again with sterile distilled water to remove excess surfactant. The seeds were then surface-sterilized by sequential treatment with 70% (*v*/*v*) ethanol for 30 s, 1.25% (*w*/*v*) sodium hypochlorite for 2 min, and finally rinsed three times with sterile distilled water to remove residual sterilant. Immature zygotic embryos were aseptically excised by carefully removing the sarcotesta layer and making a gentle incision on the embryo sac using a sterile scalpel under a stereomicroscope (Zeiss Stemi DV4, Germany).

### 4.2. Preparation of Basal Nutrient Medium

The excised embryos were cultured on three different basal media, viz., Murashige and Skoog (MS) [30], Woody Plant Medium (WPM) [31] and Chu (N6) [32], maintaining their original basal salt composition and supplemented with vitamins (Nicotinic acid; Pyridoxine HCl; Thiamine hydrochloride), amino acid (Glycine), and myo-Inositol (Table 4). Sucrose was used as the carbon source at a concentration of 30.0 gL^−1^ in MS and N6 media, while WPM was supplemented with 20.0 gL^−1^ sucrose. The pH of the MS medium was adjusted to 5.7 ± 0.1, whereas WPM and N6 were adjusted to pH 5.75 ± 0.5 using 0.1 N HCl or 0.1 N NaOH before autoclaving at 121 °C and 1.06 kg cm^−2^ pressure for 20 min. The basal media were solidified using Clarigel (HiMedia, Mumbai, India) at 2.5 gL^−1^. The required plant-growth regulators were first sterilized separately by syringe filtration through a 0.22 µm membrane filter and then added to the medium at the desired concentrations. Approximately 20 mL of medium was poured into sterile 100 mm × 15 mm Petri dishes (HiMedia, India). Later, the Petri plates were wrapped using cling film and stored at 25 ± 2 °C until further use.

### 4.3. Induction and Proliferation of Calli

For callus induction, half-strength media (MS, WPM, and N6) were supplemented with varying concentrations of auxins, specifically 2,4-dichlorophenoxyacetic acid (1.0, 1.5, 2.0, and 2.5 mgL^−1^) and picloram (1.0, 2.0, and 3.0 mgL^−1^). In addition, a control treatment comprising the respective basal media devoid of any plant-growth regulators was maintained. Cultures were maintained at 25 °C in darkness to induce callus formation. Pro-embryogenic calli emerged within 6–8 weeks and were subcultured once every 15 days onto fresh medium with the same composition to enhance callus proliferation. During each subculturing, necrotic and dark-brown tissues were carefully removed to promote healthy embryogenic growth. The number of globular and heart-shaped somatic embryos was observed using a stereomicroscope (Zeiss Stemi DV4, Jena, Germany). The experiment was conducted with eight treatments, each replicated three times (20 explants per replication), across all three basal media. Observations were made on the time required for callus initiation, callus induction percentage (%), embryogenesis percentage (%), and the nature of callus:

(1) $$Callus\;induction\;percentage\;\left(\%\right)=\frac{Number\;of\;explants\;produced\;calli\;}{Number\;of\;explants\;inoculated}\times 100$$

(2) $$Embryogenesis\;percentage\;\left(\%\right)=\frac{Number\;of\;explants\;that\;produced\;embryogenic\;callus}{Number\;of\;explants\;inoculated}\times 100$$

### 4.4. Embryo Maturation

Early-stage globular and heart-shaped somatic embryos were transferred to embryo maturation media to promote development to the cotyledonary stage. The maturation media consisted of the respective basal media supplemented with either abscisic acid (0.5, 1.0, 1.5, and 2.0 mgL^−1^) in combination with 6-benzylaminopurine (0.4 mgL^−1^) and proline (0.6 mgL^−1^) or polyethylene glycol (45, 60, 75 gL^−1^) combined with BAP (0.4 mgL^−1^) and proline (0.6 mgL^−1^). A control treatment comprising the respective basal media without the addition of abscisic acid, polyethylene glycol, BAP, or proline was also maintained. Cultures were initially maintained in the dark for 2 weeks, followed by incubation under a 16 h light and 8 h dark photoperiod for 4 weeks. Subculturing onto fresh maturation medium was performed at a 2-week interval. Somatic embryo development and progression to the cotyledonary stage were monitored periodically using a stereomicroscope (Zeiss Stemi DV4, Germany). Embryo maturation response was assessed by recording the percentage of callithose developed cotyledonary-stage embryos. Twenty embryos were used per treatment, replicated thrice across all basal media:

(3) $$Embryo\;maturation\;percentage\;\left(\%\right)=\frac{Number\;of\;calli\;at\;cotyledonary\;stage}{Number\;of\;calli\;inoculated\;at\;globular\;stage}\times 100$$

### 4.5. Plant Regeneration from Somatic Embryos and Shoot Elongation

Well-developed cotyledonary-stage somatic embryos were transferred to regeneration medium for shoot induction after six weeks of incubation in maturation medium. The regeneration media (MS, WPM, and N6) were supplemented with varying concentrations of putrescine (1.0, 1.5, 2.0, 2.5, and 3.0 mgL^−1^) and phloroglucinol (5.0 and 10.0 mgL^−1^). A control treatment comprising the respective basal media without the addition of putrescine or phloroglucinol was also included. All cultures were maintained in a plant-growth chamber under aseptic conditions (25 ± 2 °C, 80–85% relative humidity, 16/8 h photoperiod, and light intensity of 3000 lux provided by cool-white fluorescent lamps (Philips, Kolkata, India)). Subculturing was performed every 15 days, and the shoots developed after two to three subcultures, over a total period of approximately six weeks. Regeneration response was assessed after 6 weeks of culture by recording the percentage of embryos exhibiting shoot initiation, the mean shoot length (cm), and the average number of leaves per regenerated shoot. Each experiment was replicated thrice, with twenty cotyledonary embryos per replication. Somatic embryo regeneration efficiency was calculated using the following formula:

(4) $$Somatic\;embryo\;regeneration\;percentage\left(\%\right)=\frac{Number\;of\;calli\;regenerated}{Number\;of\;matured\;calli\;inoculated}\times 100$$

The regenerated plantlets were transferred to shoot elongation media based on MS, WPM, and N6 basal formulations supplemented with varying concentrations of gibberellic acid (GA_3_ @ 0, 1.0, 1.5, 2.0, 2.5, and 3.0 mgL^−1^). Cultures were subcultured at 15-day intervals to ensure sustained growth and nutrient availability. Shoot elongation response was assessed by recording the mean shoot length (cm) at each subculture stage. All cultures were maintained under controlled environmental conditions identical to those employed during the regeneration phase.

### 4.6. Root Initiation and Acclimatization

Initially, to optimize in vitro rooting, elongated shoots obtained from GA_3_-supplemented medium were cultured on media supplemented with different combinations and concentrations of auxins, viz., IAA, IBA, and NAA. The treatments included a control and various IAA (0.5 to 2.0 mgL^−1^) + NAA (0.5 or 1.0 mgL^−1^) and IBA (0.5 to 2.0 mgL^−1^) + NAA (0.5 or 1.0 mgL^−1^) combinations. These treatments were evaluated across all three half-strength basal media. Based on the rooting response, the most effective auxin combinations were further supplemented with activated charcoal (1.0 gL^−1^) and phloroglucinol (1.0 mgL^−1^) to enhance root initiation and development. Subsequently, well-developed elongated shoots (approximately 4–5 cm in height) bearing 2–3 trilobed leaves were transferred to rooting media supplemented with different auxin combinations. The treatments comprised a control and media supplemented with IAA or IBA (1.0 mgL^−1^) in combination with NAA (0.5 mgL^−1^) and phloroglucinol (1.0 mgL^−1^), with or without activated charcoal (1.0 gL^−1^). These treatments were evaluated across half-strength MS, WPM, and N6 media. The cultures were incubated for four weeks in a plant-growth chamber under controlled conditions of a 16/8 h light/dark photoperiod at 25 ± 2 °C, 80–85% relative humidity, and a light intensity of 3000 lux. After a 4-week incubation period, observations were recorded on rooting percentage (%), days taken to root, mean number of roots per shoot, and root length (cm). The experiment consisted of five treatments, with three replications, and each replication comprising 20 elongated shoots.

Plantlets with well-developed roots were gently removed from all three basal media and carefully washed under running water to remove traces of the culture medium. The plantlets were then transferred to different hardening media, viz., pot mixture (1:2:1 FYM: Red earth: Sand), cocopeat, vermicompost, and their combinations. Each pot was covered with perforated polyethylene bags to facilitate gas exchange and maintain high humidity during acclimatization. The potted plantlets were maintained at 25 ± 2 °C under 80% relative humidity with a 16 h photoperiod, and Hoagland nutrient solution was applied at five-day intervals. The survival percentage of plantlets was recorded after two months. The survival percentage was calculated using the following formula:

$$Survival\;Percentage=\;\frac{Number\;of\;plantlets\;survived\;after\;hardening}{Total\;number\;of\;plantlets\;transferred\;for\;hardening}\;\times 100$$

### 4.7. Statistical Analysis

Statistical analysis was carried out following the procedure outlined by Panse and Sukhatme [76]. The experimental design used in this research was a Factorial Completely Randomized Design (FCRD). Analysis of variance (ANOVA) was performed to determine treatment effects, and critical differences were computed at the 5% significance level. All statistical analyses were conducted in RStudio (version 2024.09.1+494, “Galactic Express”), using appropriate packages, including agricolae for mean separation and stats for ANOVA computation.

## 5. Conclusions

In the present study, a reliable and efficient in vitro regeneration protocol was established for TNAU Papaya CO 8 using immature zygotic embryos. The protocol successfully encompassed callus induction, somatic embryo development, regeneration, shoot elongation, rooting, and acclimatization, demonstrating its reproducibility and effectiveness. Among the basal media evaluated, half-strength MS medium supplemented with 2.0 mg L^−1^ 2,4-D showed the highest callus induction (81.96%) and somatic embryogenesis frequency (77.82%), while N6 medium provided moderate embryogenic responses, and WPM was comparatively less effective. Supplementation with 2.0 mg L^−1^ putrescine enhanced somatic embryo regeneration (85.02%) and shoot development, and GA_3_ (1.0 mg L^−1^) promoted vigorous shoot elongation. Efficient rooting (75.01%) and successful acclimatization (74.01%) further validated the robustness of the system. Collectively, the study demonstrates that the optimized protocol provides a highly reproducible, complete regeneration system, with MS medium offering superior performance over N6 and WPM, thereby providing a strong platform for large-scale propagation and future genetic improvement of this elite papaya variety.

## Figures and Tables

**Table 1:** Effect of 2,4-D and picloram on days taken for callus induction, callus induction percentage (%), embryogenesis percentage (%), and nature of callus from immature zygotic embryo of TNAU Papaya CO 8.

Media Strength	Treatments	Days Taken for Callus Induction (Mean ± SE)		Callus Induction Percentage (%) (Mean ± SE)		Embryogenesis Percentage (%) (Mean ± SE)		Nature of Callus
½ MS	T_1_—Control	0.00 (±0.00) ^k^		0.00 (±0.00) ^j^		0.00 (±0.00) ^i^		–
	T_2_—1.0 mgL^−1^ 2,4-D	22.20 (±1.12) ^efghi^		62.91 (±3.03) ^cd^		38.28 (±0.28) ^gh^		+
	T_3_—1.5 mgL^−1^ 2,4-D	20.98 (±0.95) ^hij^		65.01 (±0.46) ^bc^		45.02 (±2.26) ^efg^		+
	T_4_—2.0 mgL^−1^ 2,4-D	20.13 (±0.67) ^ij^		81.96 (±2.96) ^a^		77.82 (±1.3) ^a^		+++
	T_5_—2.5 mgL^−1^ 2,4-D	18.00 (±0.82) ^j^		80.20 (±2.85) ^a^		72.11 (±3.73) ^ab^		+++
	T_6_—1.0 mgL^−1^ Picloram	25.25 (±0.48) ^abcdef^		64.06 (±2.14) ^cd^		43.01 (±1.39) ^fgh^		+
	T_7_—2.0 mgL^−1^ Picloram	24.00 (±0.95) ^cdefghi^		74.09 (±3.22) ^ab^		58.07 (±0.65) ^d^		+
	T_8_—3.0 mgL^−1^ Picloram	22.07 (±0.32) ^efghi^		78.13 (±1.18) ^a^		61.00 (±1.84) ^cd^		+
½ WPM	T_1_—Control	0.00 (±0.00) ^k^		0.00 (±0.00) ^j^		0.00 (±0.00) ^i^		–
	T_2_—1.0 mgL^−1^ 2,4-D	28.15 (±1.09) ^ab^		39.91 (±0.88) ^i^		35.00 (±0.61) ^h^		+
	T_3_—1.5 mgL^−1^ 2,4-D	26.05 (±1.27) ^abcde^		44.03 (±1.9) ^ghi^		38.05 (±1.45) ^gh^		+
	T_4_—2.0 mgL^−1^ 2,4-D	24.26 (±0.48) ^bcdefgh^		54.26 (±1.85) ^def^		59.98 (±1.42) ^cd^		++
	T_5_—2.5 mgL^−1^ 2,4-D	23.19 (±0.45) ^cdefghi^		58.02 (±1.21) ^cde^		68.01 (±1.12) ^bc^		+++
	T_6_—1.0 mgL^−1^ Picloram	28.93 (±0.67) ^a^		39.93 (±1.17) ^hi^		40.27 (±1.65) ^gh^		+
	T_7_—2.0 mgL^−1^ Picloram	27.00 (±0.24) ^abc^		42.99 (±1.38) ^ghi^		54.01 (±1.51) ^de^		+
	T_8_—3.0 mgL^−1^ Picloram	26.23 (±1.12) ^abcd^		47.09 (±2.17) ^fghi^		58.08 (±2.62) ^d^		+
½ N6	T_1_—Control	0.00 (±0.00) ^k^		0.00 (±0.00) ^j^		0.00 (±0.00) ^i^		–
	T_2_—1.0 mgL^−1^ 2,4-D	24.14 (±0.27) ^cdefgh^		41.93 (±1.06) ^hi^		36.07 (±0.54) ^gh^		+
	T_3_—1.5 mgL^−1^ 2,4-D	22.95 (±1.05) ^defghi^		46.03 (±1.72) ^fghi^		42.85 (±2.23) ^gh^		+
	T_4_—2.0 mgL^−1^ 2,4-D	21.92 (±0.63) ^fghij^		55.99 (±1.84) ^cdef^		72.15 (±2.22) ^ab^		++
	T_5_—2.5 mgL^−1^ 2,4-D	21.26 (±0.54) ^ghij^		63.00 (±3.03) ^cd^		68.00 (±1.25) ^bc^		+++
	T_6_—1.0 mgL^−1^ Picloram	27.09 (±1.08) ^abc^		48.02 (±1.27) ^fghi^		51.94 (±0.36) ^def^		+
	T_7_—2.0 mgL^−1^ Picloram	26.03 (±0.44) ^abcde^		49.92 (±1.23) ^efgh^		56.01 (±2.84) ^d^		+
	T_8_—3.0 mgL^−1^ Picloram	25.06 (±0.24) ^abcdefg^		52.00 (±1) ^efg^		61.01 (±1.54) ^cd^		+
SE (d)		1.03		2.59		2.35		
CD (*p* = 0.05)		2.10		5.21		4.73		

Nature of callus was evaluated qualitatively as –: no, +: spongy (non-friable), ++: moderately friable, and +++: creamy white and friable. Values represent the mean ± standard error (SE) of three replicates (*n* = 3), and within each column, values followed by the same superscript letters are not significantly different from each other, according to the Tukey test (*p* ≤ 0.05); SE (d) = standard error of difference; CD = critical difference at 5% level. MS—Murashige and Skoog Medium; WPM—Woody Plant Medium; N6—CHU medium; 2,4–D—2,4-dichlorophenoxyacetic acid.

**Table 2:** Effect of putrescine and phloroglucinol on embryo regeneration percentage (%), with days taken for leaf emergence and shoot length (cm) on MS, WPM, and N6 media.

Media	Treatments	Embryo Regeneration Percentage (%) (Mean ± SE)		Days Taken for Leaf Emergence (Mean ± SE)		Shoot Length (cm) (Mean ± SE)	
MS	T_1_—Control	75.18 (±2.42) ^bcd^		18.01 (±0.27) ^ghi^		0.92 (±0.03) ^b^	
	T_2_—1.0 mgL^−1^ Putrescine	53.84 (±0.47) ^ghi^		32.02 (±0.65) ^abc^		0.31 (±0.01) ^ghi^	
	T_3_—1.5 mgL^−1^ Putrescine	66.01 (±2.57) ^def^		30.00 (±1.32) ^bc^		0.50 (±0.01) ^ef^	
	T_4_—2.0 mgL^−1^ Putrescine	85.02 (±4.41) ^a^		16.00 (±0.59) ^i^		1.31 (±0.03) ^a^	
	T_5_—2.5 mgL^−1^ Putrescine	78.04 (±3.46) ^ab^		21.17 (±0.2) ^efgh^		0.70 (±0.02) ^cd^	
	T_6_—3.0 mgL^−1^ Putrescine	41.98 (±0.14) ^jkl^		25.00 (±1.2) ^de^		0.40 (±0.02) ^fg^	
	T_7_—5.0 mgL^−1^ PG	0.00 (±0.00) ^m^		0.00 (±0.00) ^j^		0.00 (±0.00) ^k^	
	T_8_—10.0 mgL^−1^ PG	0.00 (±0.00) ^m^		0.00 (±0.00) ^j^		0.00 (±0.00) ^k^	
WPM	T_1_—Control	50.23 (±2.46) ^hij^		21.00 (±0.37) ^efgh^		0.60 (±0.00) ^de^	
	T_2_—1.0 mgL^−1^ Putrescine	38.00 (±0.56) ^l^		35.02 (±1.42) ^a^		0.10 (±0.01) ^jk^	
	T_3_—1.5 mgL^−1^ Putrescine	45.05 (±0.28) ^ijkl^		33.01 (±0.84) ^ab^		0.32 (±0.03) ^gh^	
	T_4_—2.0 mgL^−1^ Putrescine	57.97 (±1.01) ^fgh^		19.00 (±0.55) ^fghi^		0.70 (±0.03) ^cd^	
	T_5_—2.5 mgL^−1^ Putrescine	52.02 (±1.9) ^ghi^		23.01 (±1.17) ^ef^		0.30 (±0.02) ^ghi^	
	T_6_—3.0 mgL^−1^ Putrescine	37.01 (±0.48) ^l^		28.00 (±0.69) ^cd^		0.23 (±0.03) ^hi^	
	T_7_—5.0 mgL^−1^ PG	0.00 (±0.00) ^m^		0.00 (±0.00) ^j^		0.00 (±0.00) ^k^	
	T_8_—10.0 mgL^−1^ PG	0.00 (±0.00) ^m^		0.00 (±0.00) ^j^		0.00 (±0.00) ^k^	
N6	T_1_—Control	60.00 (±0.48) ^efg^		20.00 (±0.19) ^fghi^		0.80 (±0.03) ^c^	
	T_2_—1.0 mgL^−1^ Putrescine	48.01 (±1.7) ^ijk^		33.01 (±1.49) ^ab^		0.20 (±0.01) ^ij^	
	T_3_—1.5 mgL^−1^ Putrescine	60.00 (±0.14) ^efg^		31.00 (±1.29) ^abc^		0.40 (±0.02) ^fg^	
	T_4_—2.0 mgL^−1^ Putrescine	75.99 (±2.32) ^abc^		17.02 (±0.81) ^hi^		1.00 (±0.05) ^b^	
	T_5_—2.5 mgL^−1^ Putrescine	68.00 (±2.26) ^cde^		20.01 (±0.68) ^fghi^		0.63 (±0.04) ^d^	
	T_6_—3.0 mgL^−1^ Putrescine	40.00 (±0.47) ^kl^		22.01 (±0.77) ^efg^		0.30 (±0.01) ^ghi^	
	T_7_—5.0 mgL^−1^ PG	0.00 (±0.00) ^m^		0.00 (±0.00) ^j^		0.00 (±0.00) ^k^	
	T_8_—10.0 mgL^−1^ PG	0.00 (±0.00) ^m^		0.00 (±0.00) ^j^		0.00 (±0.00) ^k^	
SE (d)		2.40		0.63		0.03	
CD (*p* = 0.05)		4.83		1.28		0.06	

Values represent the mean ± standard error (SE) of three replicates (*n* = 3), and within each column, values followed by the same superscript letters are not significantly different from each other, according to the Tukey test (*p* ≤ 0.05); SE (d) = standard error of difference; CD = critical difference at 5% level. MS—Murashige and Skoog Medium; WPM—Woody Plant Medium; N6—CHU medium; PG—Phloroglucinol.

**Table 3:** Effect of auxins in combination with phloroglucinol and activated charcoal on rooting responses.

Media	Treatments	Rooting Percentage (%) (Mean ± SE)	Days Taken for Rooting (Mean ± SE)	Number of Roots (Mean ± SE)	Root Length (cm) (Mean ± SE)
½ MS	T_1_—Control	0.00 (±0.00) ^h^	0.00 (±0.00) ^g^	0.00 (±0.00) ^g^	0.00 (±0.00) ^h^
	T_2_—1.0 mgL^−1^ IAA + 0.5 mgL^−1^ NAA + 1.0 mgL^−1^ PG	65.01 (±0.13) ^e^	22.99 (±0.07) ^abc^	4.14 (±0.17) ^e^	2.40 (±0.02) ^f^
	T_3_—1.0 mgL^−1^ IBA + 0.5 mgL^−1^ NAA + 1.0 mgL^−1^ PG	68.14 (±1.04) ^cde^	22.23 (±0.27) ^bcd^	5.00 (±0.11) ^d^	2.80 (±0.05) ^e^
	T_4_—1.0 mgL^−1^ IAA + 0.5 mgL^−1^ NAA + 1.0 mgL^−1^ PG + 1.0 gL^−1^ AC	72.04 (±0.27) ^ab^	20.01 (±0.25) ^ef^	6.00 (±0.12) ^b^	3.41 (±0.03) ^b^
	T_5_—1.0 mgL^−1^ IBA + 0.5 mgL^−1^ NAA + 1.0 mgL^−1^ PG + 1.0 gL^−1^ AC	75.01 (±0.46) ^a^	19.02 (±0.24) ^f^	6.99 (±0.12) ^a^	3.80 (±0.03) ^a^
½ WPM	T_1_—Control	0.00 (±0.00) ^h^	0.00 (±0.00) ^g^	0.00 (±0.00) ^g^	0.00 (±0.00) ^h^
	T_2_—1.0 mgL^−1^ IAA + 0.5 mgL^−1^ NAA + 1.0 mgL^−1^ PG	54.04 (±1.04) ^g^	23.65 (±0.18) ^ab^	3.40 (±0.04) ^f^	2.10 (±0.02) ^g^
	T_3_—1.0 mgL^−1^ IBA + 0.5 mgL^−1^ NAA + 1.0 mgL^−1^ PG	59.17 (±1.02) ^f^	23.03 (±0.22) ^abc^	4.18 (±0.05) ^e^	2.42 (±0.08) ^f^
	T_4_—1.0 mgL^−1^ IAA + 0.5 mgL^−1^ NAA + 1.0 mgL^−1^ PG + 1.0 gL^−1^ AC	65.29 (±0.35) ^de^	22.01 (±0.22) ^cd^	5.02 (±0.11) ^d^	3.02 (±0.08) ^d^
	T_5_—1.0 mgL^−1^ IBA + 0.5 mgL^−1^ NAA + 1.0 mgL^−1^ PG + 1.0 gL^−1^AC	69.00 (±1.29) ^bcd^	21.02 (±0.32) ^de^	5.50 (±0.09) ^c^	3.19 (±0.06) ^cd^
½ N6	T_1_—Control	0.00 (±0.00) ^h^	0.00 (±0.00) ^g^	0.00 (±0.00) ^g^	0.00 (±0.00) ^h^
	T_2_—1.0 mgL^−1^ IAA + 0.5 mgL^−1^ NAA + 1.0 mgL^−1^ PG	60.12 (±1.18) ^f^	24.00 (±0.51) ^a^	3.01 (±0.01) ^f^	2.30 (±0.00) ^fg^
	T_3_—1.0 mgL^−1^ IBA + 0.5 mgL^−1^ NAA + 1.0 mgL^−1^ PG	65.29 (±0.4) ^de^	23.00 (±0.35) ^abc^	4.51 (±0.01) ^e^	2.50 (±0.03) ^f^
	T_4_—1.0 mgL^−1^ IAA + 0.5 mgL^−1^ NAA + 1.0 mgL^−1^ PG + 1.0 gL^−1^ AC	70.02 (±0.46) ^bc^	21.00 (±0.42) ^de^	5.50 (±0.1) ^c^	3.27 (±0.07) ^bc^
	T_5_—1.0 mgL^−1^ IBA + 0.5 mgL^−1^ NAA + 1.0 mgL^−1^ PG + 1.0 gL^−1^ AC	72.01 (±0.98) ^ab^	19.99 (±0.42) ^ef^	6.09 (±0.11) ^b^	3.40 (±0.03) ^bc^
SE (d)		1.033	0.394	0.123	0.042
CD (*p* = 0.05)		2.11	0.805	0.252	0.121

Values represent the mean ± standard error (SE) of three replicates (*n* = 3), and within each column, values followed by the same superscript letters are not significantly different from each other, according to the Tukey test (*p* ≤ 0.05); SE (d) = standard error of difference; CD = critical difference at 5% level. MS—Murashige and Skoog Medium; WPM—Woody Plant Medium; N6—CHU medium; IAA—Indole-3-acetic acid; NAA—Naphthalene acetic acid; IBA—Indole-3-butyric acid; PG—Phloroglucinol; AC—Activated charcoal.

**Table 4:** Media composition of Murashige and Skoog (MS), Woody Plant Medium (WPM), and Chu (N6) media.

Ingredients	MS Medium (mgL^−1^)	WPM (mgL^−1^)	CHU (N6) Medium (mgL^−1^)
Major elements			
Ammonium nitrate	1650.00	400.00	463.00
Potassium nitrate	1900.00	-	2830.00
Magnesium sulphate	180.70	180.69	90.37
Potassium phosphate monobasic	170.00	170.00	400.00
Calcium chloride	332.20	72.50	125.34
Calcium nitrate monohydrate	-	386.34	-
Potassium sulphate	-	990.00	-
Minor elements			
Manganese sulphate	16.90	22.30	3.33
Zinc sulphate heptahydrate	8.60	8.60	1.50
Boric acid	6.20	6.20	1.60
Molybdic acid (sodium salt)	0.25	0.213	-
Copper sulphate pentahydrate	0.025	0.025	-
Cobalt chloride	0.025	-	-
Disodium EDTA dihydrate	37.26	37.30	37.30
Ferrous sulphate heptahydrate	27.8	27.80	27.80
Potassium Iodide	0.83	-	0.80
Vitamins			
Myo-Inositol	100.00	100.00	-
Nicotinic acid (free acid)	0.50	0.50	0.50
Pyridoxine HCl	0.50	0.50	0.50
Thiamine hydrochloride	0.10	1.00	1.00
Amino acid			
Glycine	2.00	2.00	2.00
Carbohydrate			
Sucrose	30.00 gL^−1^	20.00 gL^−1^	30.00 gL^−1^
pH	5.7 ± 0.1	5.75 ± 0.5	5.75 ± 0.5

## Data Availability

The original contributions presented in this study are included in the article. Further inquiries can be directed to the corresponding authors.

## Abbreviations

The following abbreviations are used in this manuscript:

2,4-D	2,4-dichlorophenoxyacetic acid
ABA	Abscisic acid
BAP	6-benzylaminopurine
GA_3_	Gibberellic acid
PG	Phloroglucinol
IAA	Indole-3-Acetic Acid
IBA	Indole-3-butyric acid
NAA	Naphthalene Acetic Acid
MS	Murashige and Skoog
WPM	Woody Plant Medium
N6	CHU
AC	Activated Charcoal
LEA	Late Embryogenesis Abundant
FYM	Farm Yard Manure
